# Mutation analysis performed on tumor biopsies from patients with newly-diagnosed germinal center aggressive B cell lymphomas

**DOI:** 10.18632/oncotarget.28309

**Published:** 2022-11-17

**Authors:** Daniel J. Landsburg, Jennifer J.D. Morrissette, Stephen J. Schuster, Sunita D. Nasta, James N. Gerson, Stefan K. Barta, Jakub Svoboda, Elise A. Chong, Megan S. Lim

**Affiliations:** ^1^Department of Medicine, University of Pennsylvania, Philadelphia, PA 19104, USA

**Keywords:** diffuse large B cell lymphoma, high grade B cell lymphoma, mutation analysis, next generation sequencing, chemotherapy

## Abstract

Comprehensive genomic analyses of tumor biopsies from patients with newly-diagnosed germinal center B cell (GCB) diffuse large B cell/high grade B cell lymphoma (DLBCL/HGBL) have identified molecular subtypes predictive of inferior survival, which are characterized by somatic mutations that can be detected through clinical laboratory mutation analysis (CLMA). To determine the frequency and predictive value of individual genetic mutations associated with these experimentally-defined poor-risk subgroups, we reviewed the findings from CLMA performed on tumors from patients with newly-diagnosed GCB DLBCL/HGBL who were previously treated at our institution. CLMA was successfully performed on 58/59 patient tumor biopsies with a median turnaround time of 16 days, and 51 on which CLMA was routinely performed with adequate clinical follow-up were analyzed. Patients whose tumors demonstrated CREBBP mutation experienced a lower estimated rate of 2-year disease free survival (DFS) as compared to those whose tumors did not (45% [95% CI 18–68%] vs. 67% [95% CI 44–83%], *P* = 0.045). CREBBP mutations may be frequent and predict for inferior DFS in patients with newly-diagnosed GCB DLBCL/HGBL. Furthermore, CLMA may be practically-applied to translate experimental findings into those with more direct application to risk stratification and clinical trial design in subsets of patients with DLBCL/HGBL.

## INTRODUCTION

While patients diagnosed with germinal center B (GCB) cell of origin (COO) diffuse large B cell lymphoma (DLBCL)/high grade B cell lymphoma (HGBL) may experience more favorable survival outcomes following receipt of first-line rituximab, cyclophosphamide, doxorubicin, vincristine and prednisone (R-CHOP) as compared to those diagnosed with activated B cell (ABC) COO DLBCL/HGBL [1], comprehensive genomic analyses have revealed that tumors from approximately 50% of newly-diagnosed GCB DLBCL/HGBL patients can be assigned to a poor-risk subgroup (Cluster 3, EZB) which is associated with inferior survival following receipt of first line R-CHOP [2, 3]. Additionally, 10–25% of tumors from newly-diagnosed GCB DLBCL/HGBL patients demonstrate poor-risk gene expression profiles which assign them to a subgroup (double hit signature, molecular high grade) which is also associated with inferior survival following receipt of first line R-CHOP [4, 5].

These poor-risk GCB DLBCL/HGBL subgroups are characterized by recurring genetic mutations, which can be detected by clinical laboratory mutation analysis (CLMA). Thus, we sought to analyze the results of CLMA performed at our institution on tumors from patients with newly-diagnosed GCB DLBCL/HGBL previously-treated with first line immunochemotherapy to determine the frequency and predictive value of the presence of individual genetic mutations associated with these experimentally-defined poor-risk subgroups.

## RESULTS

**Figure 1:**
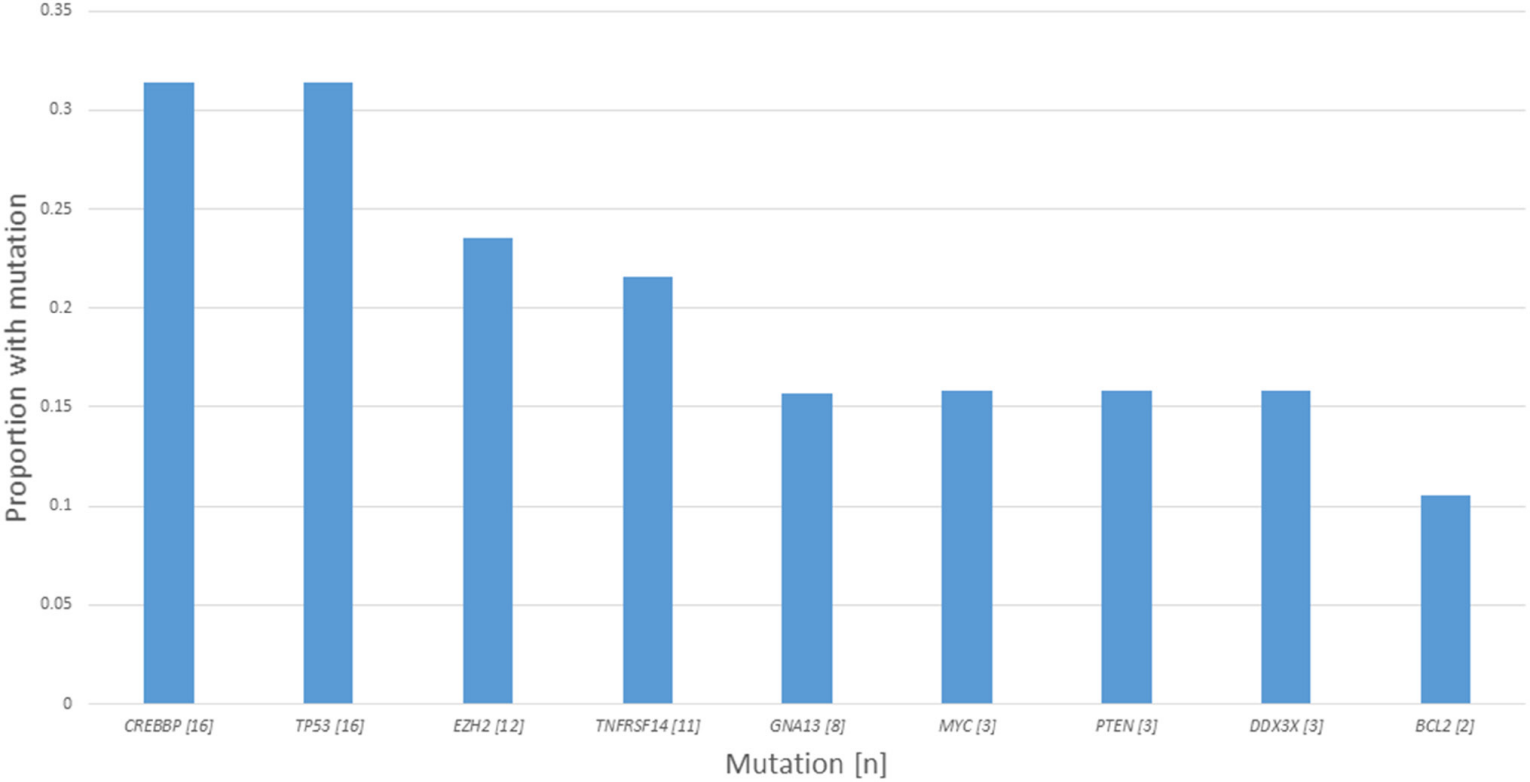
Number and frequency of detected mutations of interest.

CLMA was performed on 59 tumor biopsies (48 formalin-fixed paraffin-embedded tissue, 11 bone marrow aspirate or biopsy, 2 body fluid) obtained from 2015–21, with 58 successful assays (98% success rate) and a median result turnaround time (TAT) of 16 days. Five biopsies were excluded due to documented request by treating clinician (3) or diagnosing pathologist (2) for purposes of medical decision making and 2 additional biopsies were excluded due to lack of clinical follow-up, resulting in analysis of 51 biopsies from 51 patients, for which Lymphoma Sequencing Panel (LSP) was performed on 32 and PennSeq™ Lymphoma Panel PSLP on 19. Baseline clinicopathologic characteristics are listed in Table 1 and a histogram of detected mutation number/frequency in Figure 1. Of note, 46 specimens expressed CD10 by IHC or flow cytometry and 35 harbored at least one mutation in a gene of interest. All patients treated with intensive immunochemotherapy (*n* = 15) received rituximab, etoposide, prednisone, vincristine, cyclophosphamide and doxorubicin (R-EPOCH).

**Table 1:** Baseline characteristics

Characteristic	*n* (%)
**Median age**	65 years
**International Prognostic Index Score**	
**<3**	28 (55)
**≥3**	23 (45)
**Histology**	
**DLBCL**	43 (84)
**HGBL**	8 (16)
**Transformed indolent lymphoma**	
**No**	43 (84)
**Yes**	8 (16)
**MYC IHC**	
**<40%**	23 (45)
**≥40%**	21 (41)
**Unknown**	7 (14)
**Double expressor lymphoma**	
**No**	35 (69)
**Yes**	10 (20)
**Unknown**	6 (11)
***MYC* rearrangement by FISH **	
**No**	40 (78)
**Yes**	10 (20)
**Unknown**	1 (2)
**Double hit lymphoma**	
**No**	45 (88)
**Yes**	5 (10)
**Unknown**	1 (2)

**Figure 2:**
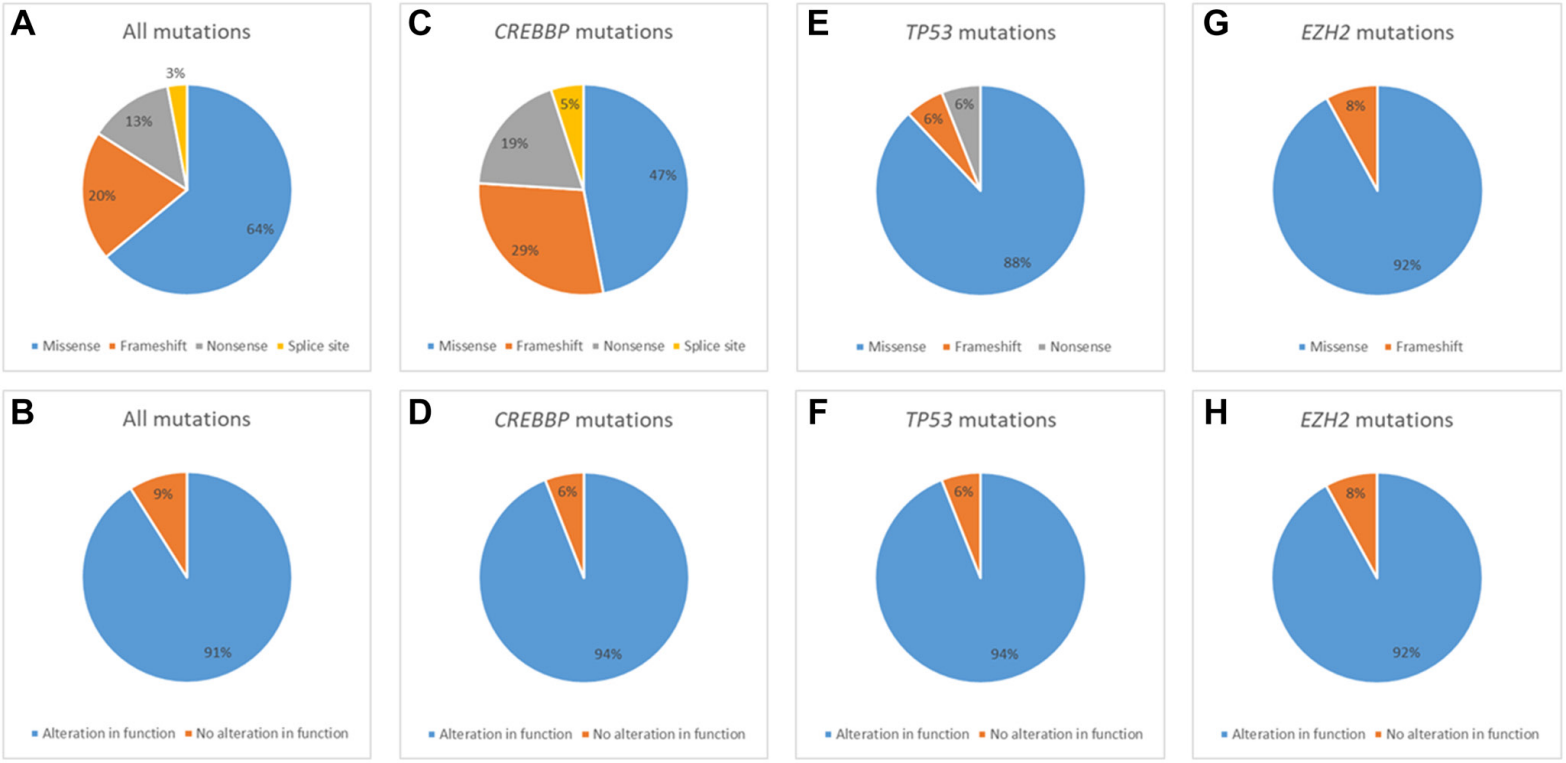
Mutation type and predicted impact of mutation on alteration of gene function for all mutations (**A**, **B**), CREBBP mutations (**C**, **D**), TP53 mutations (**E**, **F**) and EHZ2 mutations (**G**, **H**).

**Figure 3:**
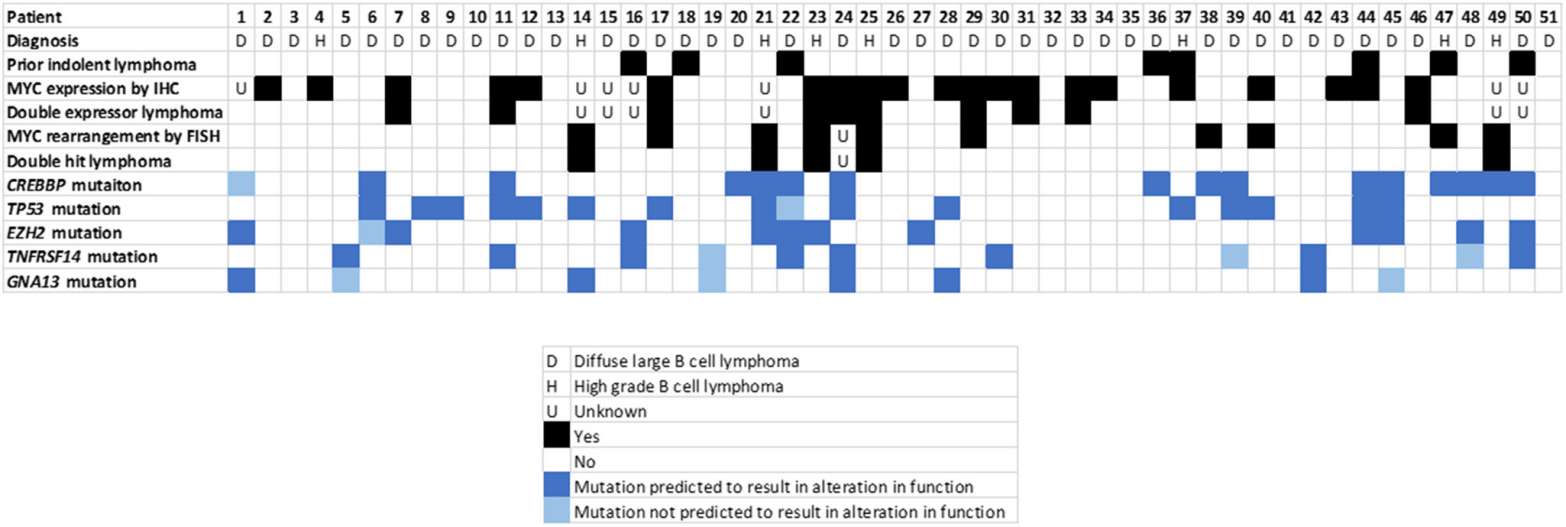
Tumor characteristics analyzed, by patient.

For all 51 biopsies analyzed, there were a total of 87 mutations characterized as 56 missense, 17 frameshift, 11 nonsense and 3 splice site, and 35 biopsies harbored a mutation of a gene of interest with 32 biopsies a mutation of a gene of interest with gain or loss of function predicted. The median number of mutations of genes of interest was 1 (range 0–6) and mutations of genes of interest with gain or loss of function predicted was 1 (range 0–4). In total, there were 74 occurrences of mutations of genes of interest (counting duplicate mutations in the same gene in the same biopsy only once), with 60 predicted to result in gain or loss of gene function. For *CREBBP*, 21 mutations were characterized as 10 missense, 6 frameshift, 4 nonsense and 1 splice site with loss of function predicted to result in 15/16 biopsies. For *TP53*, 18 mutations were characterized as 16 missense, 1 frameshift and 1 nonsense with loss of function predicted to result in 15/16 biopsies. For *EZH2*, 12 mutations were characterized as 11 missense and 1 frameshift with gain of function predicted to result in 11/12 biopsies. For *TNFRSF14*, 11 mutations were characterized as 2 missense, 5 frameshift, 3 nonsense, 1 splice site with loss of function predicted to result in 8/11 biopsies. For other genes of interest (*GNA13, BCL2, DDX3X, MYC* and *PTEN*), 24 mutations were characterized as 17 missense, 4 frameshift, 2 nonsense and 1 splice site with gain or loss of function predicted in 11/19 biopsies. A summary of mutation characteristics is depicted in Figure 2 and a summary of tumor characteristics by patient is depicted in Figure 3.

**Figure 4:**
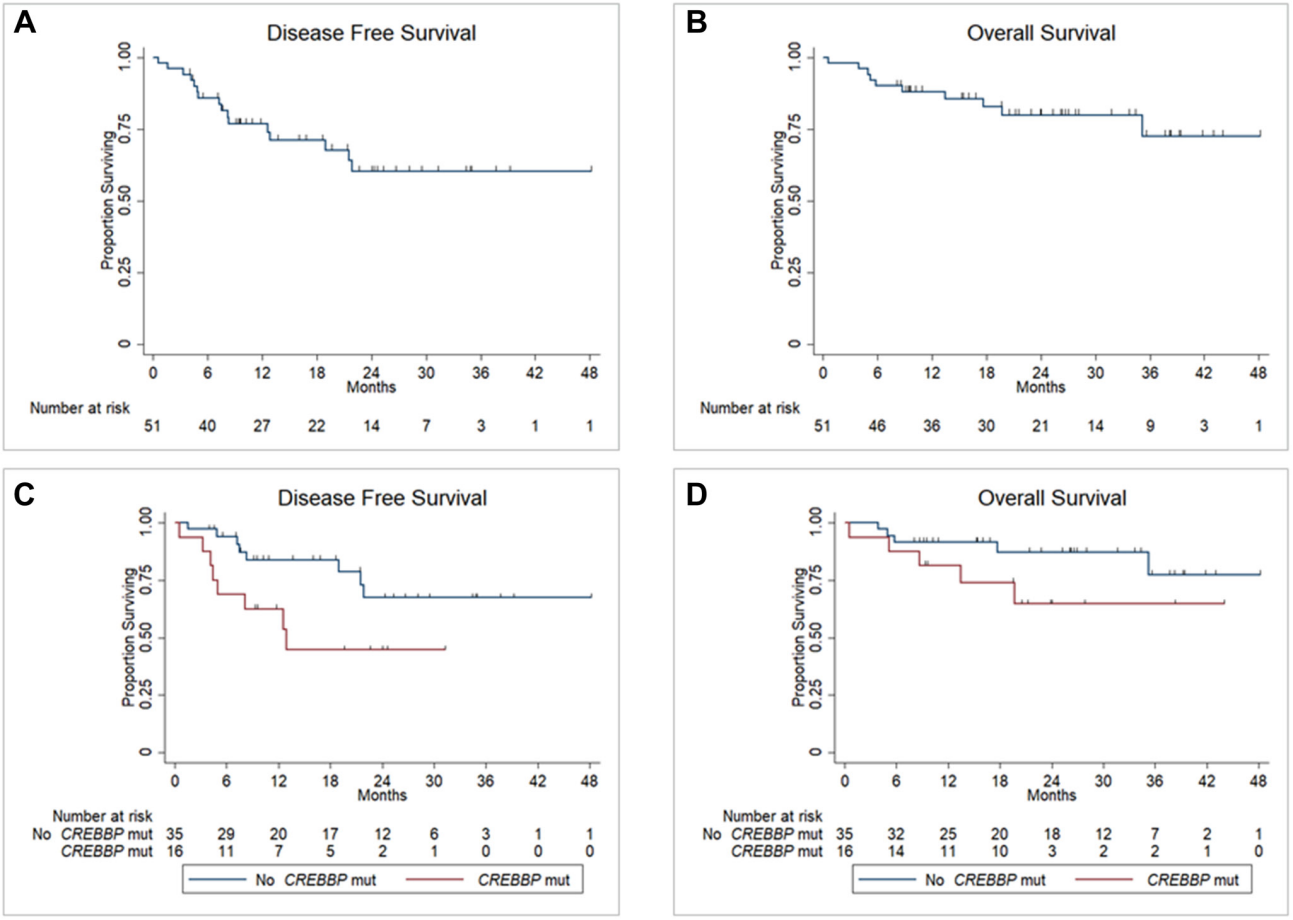
Disease free survival (**A**) and overall survival (**B**) for all patients; disease free survival (**C**) and overall survival (**D**) for all patients by CREBBP mutation status. Abbreviation: Mut: mutation.

With a median follow-up of 25.2 months, the Kaplan Meier estimate of 2 year (y) DFS was 60% (95% confidence interval [CI] 41–74%) and 2y OS 80% (95% CI 64-89%) for all patients as depicted in Figure 4A and 4B, respectively. Characteristics listed in Table 1 as well as mutations detected in both LSP and PSLP predictive of disease relapse at 2y with *P* < 0.10 by univariate analysis were International Prognostic Index (IPI) score ≥3 vs. <3 (hazard ratio [HR] 2.3, 95% CI 0.86–6.5, *P* = 0.098), HGBL vs. DLBCL histology (HR 5.4, 95% CI 1.8–15.7, *P* = 0.002), double hit lymphoma vs. not (HR 9.0, 95% CI 2.6–30.8, *P* < 0.001) and *CREBBP* mutation vs. not (HR 2.6, 95% CI 0.98–7.0, *P* = 0.054). Univariate Cox regression analysis for death at 2 years performed for these factors revealed IPI score ≥3 vs. <3 (hazard ratio [HR] 11.3, 95% CI 1.4–90.5, *P* = 0.002), HGBL vs. DLBCL histology (HR 5.0, 95% CI 1.3–18.6, *P* = 0.02), double hit lymphoma vs. not (HR 8.0, 95% CI 1.8–36.0, *P* = 0.007) and *CREBBP* mutation vs. not (HR 2.8, 95% CI 0.76–10.5, *P* = 0.12). A meaningful multivariate analysis for factors predictive of disease relapse or death could not be carried out due to small sample size.

Estimated 2y DFS was significantly lower for patients whose tumors demonstrated *CREBBP* mutation vs. not (45% [95% CI 18–68%] vs. 67% [95% CI 44–83%], *P* = 0.045) as depicted in Figure 4C, but not based upon the presence or absence of other mutations analyzed. Estimated 2y OS for patients whose tumors demonstrated *CREBBP* mutation vs. not did not differ significantly (65% [95% CI 34–84%] vs. 87% [95% CI 68–95%], *P* = 0.11) as depicted in Figure 4D. All patients with *CREBBP* mutation who relapsed did so ≤12 months from diagnosis. Characteristics analyzed from Table 1 and co-mutation frequency did not differ significantly when comparing patients whose tumors demonstrated *CREBBP* mutation vs. not, with the exception of *EZH2* co-mutation (50% with *CREBBP* mutation vs. 11% without *CREBBP* mutation, *P* = 0.005). Estimated 2y DFS did not differ significantly for patients treated with R-CHOP (*n* = 36) as compared to R-EPOCH (*n* = 15) in the entire cohort (61% [95% CI 37–78%] vs. 58% [95% CI 29–79%], *P* = 0.72), nor for those whose tumors demonstrated *CREBBP* mutations (*n* = 12 vs. *n* = 4, 53% [95% CI 20–78%] vs. 25% [95% CI 9–67%], *P* = 0.42).

## DISCUSSION

Our analysis demonstrates that the presence of *CREBBP* mutations in tumor biopsies from patients with newly-diagnosed GCB DLBCL/HGBL is associated with poorer DFS following treatment with front-line immunochemotherapy. *CREBBP* encodes an acetyltransferase protein which is a transcription factor responsible for several cellular functions including activation of p53 and repression of BCL6 [6], and therefore *CREBBP* mutations resulting in loss of function can promote lymphomagenesis. A high frequency of *CREBBP* mutations has also been demonstrated in biopsies from newly-diagnosed DLBCL/HGBL patients undergoing large-scale mutation analysis from the SAAK 38/07 prospective clinical trial cohort [7] as well as those treated in the GOYA study [8], although the predictive value of *CREBBP* mutations for DFS in GCB DLBCL/HGBL patients was not clearly stated in either analysis.

In terms of other mutations analyzed, the presence of *TP53* mutations has been associated with a poor prognosis for patients diagnosed with lymphoid malignancies, including those with newly diagnosed GCB DLBCL treated with R-CHOP [9]. While our analysis does not support this finding, 2y DFS was 42% for patients with *TP53* mutation with *CREBBP* co-mutation vs 63% for patients with *TP53* mutation without *CREBBP* co-mutation analyzed in our series. This raises the possibility that *TP53* mutations may predict for poorer DFS only in the presence of *CREBBP* co-mutation, perhaps due to a dual impact on *TP53* transcription by inhibition of acetylation-driven *TP53* activation mediated by loss-of-function *CREBBP* mutations as well as direct damage to *TP53* mediated by loss-of-function *TP53* mutations. Additionally, the other commonly detected mutated genes in our series, *EZH2* and *TNFRS14*, were also not associated with DFS in patients with GCB DLBCL treated in the GOYA study [8].

Strengths of our analysis include a moderate sample size of biopsies on which CLMA was performed routinely without known selection bias at a single center on tumors from patients with comparable baseline characteristics to those of larger unselected DLBCL patient cohorts, including 45% with IPIs score ≥3 [10], 20% with double expressor lymphoma [11] and 10% with double hit lymphoma [12], which suggests that our finding may be applicable to the general population of newly-diagnosed GCB DLBCL/HGBL patients. Additionally, we demonstrate the high success rate and rapid result turnaround time of CLMA, which implies that results from this assay could be feasibly be incorporated into initial management decisions for these patients.

Weaknesses of our analysis include the use of two sequencing panels over time with one panel not detecting mutations in *BCL2, DDX3X, MYC* and *PTEN* and neither in *KTM2D*, both which limited the ability to detect potential survival differences based on the detection of mutations in all genes of interest. However, at least *MYC* mutations [13] nor *BCL2* mutations [14] have been clearly associated with inferior survival outcomes in patients with newly-diagnosed GCB DLBCL/HGBL treated with R-CHOP. Additionally, patients were not treated with a uniform first-line therapy, although the result of the CALGB/Alliance 50303 trial demonstrated no difference in survival for patients with newly diagnosed DLBCL/HGBL receiving R-CHOP as compared to dose-adjusted R-EPOCH [15].

In conclusion, CLMA performed on tumor biopsies from patients with newly-diagnosed GCB DLBCL/HGBL revealed frequent mutations in *CREBBP* which were predicted to result in loss of function as well as a significantly lower rate of estimated DFS at 2 years. These findings support efforts to confirm the predictive value of *CREBBP* mutations in a larger cohort of newly-diagnosed GCB DLBCL/HGBL patients as well investigation of agents such as histone deacetylase inhibitors which may overcome loss of histone acetyltransferase function in patients with *CREBBP*-mutated GCB DLBCL/HGBL. Furthermore, we have reported the first known attempt to translate findings from experimental molecular assays through CLMA in order to identify the frequency and predictive value of individual gene mutations within a specific DLBCL/HGBL patient population, and our results support future efforts of this type given their potential to practically inform DLBCL/HGBL patient risk stratification as well as the design of future clinical trials incorporating agents targeted against specific gene mutations.

## MATERIALS AND METHODS

Inclusion criteria for this analysis were diagnosis of GCB DLBCL/HGBL (either *de novo* or transformed indolent lymphoma but without receipt of prior cytotoxic chemotherapy), receipt of either R-CHOP or an intensive first-line immunochemotherapy and performance of mutation analysis with one of two lymphoma-specific gene sequencing panels at the Penn Center for Personalized Diagnostics at the University of Pennsylvania (Lymphoma Sequencing Panel [LSP] from 2018–20 and PennSeq™ Lymphoma Panel [PSLP] from 2020–22). Exclusion criteria included history of chronic lymphocytic leukemia, clinician or pathologist request for mutation analysis and lack of adequate follow-up.

LSP and PSL were designed to detect single nucleotide variants (SNVs), insertions and deletions (indels), with a minimum tumor percentage of 10% tumor cells. Acceptable specimens are blood, bone marrow, fine needle aspirations and formalin fixed paraffin embedded tissue. DNA was extracted using the Agencourt FormaPure kit (PET, Beckman Coulter, Brea, CA, USA) or the DSP Mini Kit or Gentra PureGene Blood Kit for low volume samples (blood, bone marrow, Qiagen, Germantown, MD, USA). LSP is an amplicon based Next Generation Sequencing (NGS) oncology panel designed to target genes recurrently mutated in lymphomas. A minimum input of 10 ng of DNA was required for the Lymphoma 40 Kit (Illumina, Carlsbad, CA, USA). Libraries were prepared based on manufacturers instruction to target 40 genes with SNVs and indels called at 5% VAF in the following genes: ATM, B2M, BIRC3, BRAF, BTK, CARD11, CD79A, CD79B, CIITA, CREBBP, CXCR4, EGR2, EZH2, GNA13, ID3, IDH2, JAK3, KLF2, MAP2K1, MYD88, NFKBIE, NOTCH1, NOTCH2, PLCG1, PLCG2, POT1, RHOA, RPS15, RRAGC, SF3B1, SOCS1, STAT3, STAT5B, TCF3, TET2, TNFAIP3, TNFRSF14, TP53, TRAF3, and XPO1. Sequencing of the genes in bold included the entire coding region. Sequencing of the pooled sequencing libraries took place on the MiSeq (Illumina, Carlsbad, CA, USA), with fastqs run through a custom bioinformatics pipeline, were reviewed and reported using HGVS nomenclature (http://varnomen.hgvs.org/). All variants are reported based on the hg19 genome build. PSLP is a custom hybrid-capture-based Next Generation Sequencing (NGS) oncology panel. A minimum input of 100 ng for DNA derived from non-FFPE or 200 ng of DNA derived from FFPE DNA is sheared and then prepped into a whole-genome library. The library is target-enriched using capture probes covering exonic regions of 502 gene targets, a 9,000 SNP backbone and additional probes for biomarker detection, with 116 genes reported for SNVs, indels and limited copy number calling: *ABL1*, *ASXL1*, *ATM*, *B2M*, *BCL2*, *BCOR*, *BCORL1*, *BIRC3*, *BRAF*, *BRCA1*, *BRCA2*, *BRIP1*, *BRINP3*, *BTK*, *CALR*, *CARD11*, *CBL*, *CD79A*, *CD79B*, *CDKN2A*, *CEBPA*, *CIITA*, *CREBBP*, *CSF1R*, *CSF3R*, *CXCR4*, *DDX3X*, *DDX41*, *DICER1*, *DNMT3A*, *EGR2*, *ERCC4*, *ETV6*, *EZH2*, *FANCA*, *FANCC*, *FANCD2*, *FANCE*, *FANCF*, *FANCG*, *FANCL*, *FANCM*, *FBXW7*, *FLT3*, *GATA2*, *GNA13*, *GNAS*, *HNRNPK*, *ID3*, *IDH1*, *IDH2*, *IKZF1*, *IL7R*, *JAK2*, *JAK3*, *KIT*, *KLF2*, *KLHL6*, *KRAS*, *MAP2K1*, *PAK1*, *RIP142*, *MPL*, *MYC*, *MYCN*, *MYD88*, *NF1*, *NFKBIE*, *NOTCH1*, *NOTCH2*, *NPM1*, *NRAS*, *PALB2*, *PDGFRA*, *PHF6*, *PLCG1*, *PLCG2*, *POT1*, *PRPF40B*, *PTEN*, *PTPN11*, *RAD21*, *RAD51*, *RAD51C*, *RHOA*, *RIT1*, *RPS15*, *RRAGC*, *RUNX1*, *SETBP1*, *SF1*, *SF3A1*, *SF3B1*, *SLX4*, *SMC1A*, *SOCS1*, *SRSF2*, *STAG2*, *STAT3*, *STAT5B*, *TBL1XR1*, *TCF3*, *TERT*, *TET2*, *TNFAIP3*, *TNFRSF14*, *TP53*, *TPMT*, *TRAF3*, *U2AF1*, *U2AF2*, *WT1*, *XPO1*, *XRCC2*, *ZMYM3*, *ZRSR2.* Sequencing of the pooled sequencing libraries took place on the NovaSeq (Illumina, Carlsbad, CA, USA), with fastqs run through a custom bioinformatics pipeline, were reviewed and reported using HGVS nomenclature (http://varnomen.hgvs.org/). All variants are reported based on the hg38 genome build. Gene mutations were further characterized by structural type and effect on gene function was primarily determined through the OncoKB [16] and PolyPhen-2 [17] databases as well as primary literature when available as referenced for individual mutations [9, 18].

Based on the aforementioned publications, genetic mutations of interest were *BCL2, CREBBP, DDX3X, EZH2, GNA13, KMT2D, MYC, PTEN TNFRSF14* and *TP53*; however, *BCL2, DDX3X, MYC* and *PTEN* were only available in the PSLP and *KTM2D* was not available in either panel. Institutional standards for pathologic evaluation of tumor biopsies included immunohistochemical staining (IHC) for CD10, BCL6, MUM1, MYC and BCL2 among other makers, with cell of origin assigned by Hans algorithm, [19] as well as fluorescence *in situ* hybridization (FISH) for *MYC* rearrangement with reflex testing for *BCL2* and *BCL6* rearrangement if positive. Therapy was given at the discretion of the treating physician. Disease free survival (DFS) was defined as the interval between diagnosis of DLBCL/HGBL to DLBCL/HGBL relapse or last follow-up in remission. Overall survival (OS) was defined as the interval between diagnosis and time of death or last follow-up while alive. Data were censored on 7/1/22. Disease response by computed tomography with or without positron emission tomography was determined by the Revised Response Criteria for Malignant Lymphoma.[20] Survival curves were plotted using Kaplan-Meier estimates, and survival analysis was performed using the log-rank test. Univariate analysis was performed using Cox proportional-hazards regression. Categorical variables were analyzed by Fisher’s exact test. Statistical significance was defined as a two-tailed *P* value < 0.05 unless otherwise specified. All statistical analyses were performed using Stata version 13 (StataCorp, College Station, TX, USA). This protocol was approved by the Institutional Review Board of the University of Pennsylvania.
